# Association of Expanded Health Care Networks With Utilization Among Veterans Affairs Enrollees

**DOI:** 10.1001/jamanetworkopen.2021.31141

**Published:** 2021-10-26

**Authors:** Liam Rose, Marion Aouad, Laura Graham, Lena Schoemaker, Todd Wagner

**Affiliations:** 1 Health Economics Resource Center, Veterans Affairs Palo Alto Health Care System, Menlo Park, California; 2 Stanford Surgery Policy Improvement Research and Education Center, Stanford School of Medicine, Stanford, California; 3 Department of Economics, University of California, Irvine

## Abstract

**Importance:**

Health insurers alter the size of their networks, offering lower premiums in exchange for a more limited set of care choices. However, little is known about the association of network size with health care utilization and outcomes, particularly outside of the context of private insurance plans.

**Objective:**

To evaluate changes in health care utilization after an expansion in the Veterans Affairs Health Care System (VA) health care network.

**Design, Setting, and Participants:**

This cross-sectional study included individuals enrolled in the VA from 2015 to 2018. Considering that the health care network expansion only affected a portion of enrollees, only those who lived between 20 and 60 miles from a VA facility were included. Data analysis was conducted from September 2020 to February 2021.

**Exposures:**

Individuals who lived 40 or more miles away from a VA facility were automatically eligible for an expanded health care network through non-VA practitioners (VA community care); those living less than 40 miles away from a VA facility were not automatically eligible.

**Main Outcomes and Measures:**

A regression discontinuity analysis of individuals who became eligible for an expanded network based on geographic residence was performed. Inpatient and outpatient utilization rates per VA enrollee during the study period, with utilization differentiated by whether services were provided by a VA or non-VA practitioner, were calculated.

**Results:**

The study included more than 2.7 million unique individuals whose characteristics largely reflected the demographic characteristics of the VA system (mean [SD] age, 62 [17] years; 2 589 252 [90%] men; 282 168 [10%] Black; 2 203 352 [77%] White). Patient characteristics (age, race, and comorbidities) did not vary significantly by eligibility status. Outpatient utilization was 3.2% higher (95% CI, 1.0% to 5.3%) among those with access to an expanded network. Increased utilization was most pronounced among those with a higher VA disability rating (3.1%; 95% CI, 0.5% to 5.7%) and among younger individuals without service-connected disabilities (7.0%, 95% CI, 1.7% to 12.3%). There was no evidence of changes to inpatient utilization (1.2%; 95% CI. –2.5% to 4.9%; *P* = .37) for those with access to the expanded network.

**Conclusions and Relevance:**

In this study, expanded network access was associated with increased total health care utilization among affected enrollees in the VA. Understanding how network size affects utilization is immediately informative for the VA, but it can also help to guide policies for insurance markets.

## Key Points

**Question:**

Are expanded health care options associated with inpatient and outpatient utilization rates?

**Findings:**

In this cross-sectional study of more than 2.7 million Veterans Health Administration enrollees, expanding health care networks was associated with higher outpatient utilization.

**Meaning:**

These findings suggest that larger health care networks are associated with more intensive health care use by affected patients.

## Teaser

This cross-sectional study evaluates the change in health care utilization among enrollees in the Veteran Affairs Health Care System (VA) after an expansion in the covered network.

## Introduction

Health insurers use selective contracting to create a network of clinicians, hospitals, and health care systems.^1,2^ Creating narrow networks can lead to decreases in utilization, which results in lower costs.^3^ Narrowing is typically done by excluding higher-priced practitioners, but it can also be used to exclude those who deliver care to more vulnerable and less profitable patients.^4,5^ With the growth of so-called skinny networks, recent policy debate has focused on how narrower networks affect consumers.

When faced with an option to join a narrow network with lower premiums, many individuals are willing to pay higher premiums to maintain continuity with their clinicians.^6,7^ Those who choose an insurance plan with a narrow network may see lower premiums and expenditures, but it can also leave them with fewer within-network options. For these individuals, narrow networks can become costly, both by way of increased immediate financial costs if they visit out-of-network practitioners and by way of worsened health outcomes due to disruptions in continuity of care or decreased access to care.

To our knowledge, the research to date has focused on estimating the effect of narrowing networks. However, examining network expansions is also informative because it can provide an alternative and complementary way to examine the impacts of insurance network changes on an individual’s health care decisions and outcomes. To that end, this study asks, what is the association of expanded health care options with individuals’ health care choices and outcomes?

To examine this question, we leveraged the introduction of the Veterans Choice Act. In 2014, Congress enacted the Veterans Access, Choice, and Accountability Act, commonly referred to as the Choice Act.^8^ This program was enacted in response to reports of excessive wait times and inadequate access in the Department of Veterans Affairs (VA) health network. Prior to the enactment of this policy, many veterans could only access non-VA health care practitioners if the necessary services were not available through the VA or if the individual had private insurance, Medicaid, or Medicare. Thus, with the passage of the program, veterans who were eligible to receive health care through the VA were now also eligible to receive health care in non-VA facilities (ie, the community) if they met at least 1 of the eligibility criteria. Importantly, the Choice Act did not affect patient copayments or cost-sharing arrangements, whether the care was received at the VA or in the community.

This study compares health care utilization and mortality of individuals eligible for the Choice Act with those who were not eligible. We used a regression discontinuity (RD) design because of the sharp cutoff in eligibility for community care through the Choice Act. In particular, the RD design compares health outcomes for individuals who lived at a distance just greater than the arbitrarily set threshold (ie, 40 miles) with individuals living at a distance less than this threshold. This estimation strategy identifies how the program was associated with individuals’ health care decisions and outcomes. In turn, this allows us to identify how individuals respond to expanded health care access.

## Methods

For our study, we included all individuals who were eligible to receive health care through the VA. We specifically examined data for the years 2015 to 2018, which includes approximately 10.8 million people. During this time, approximately 2 million enrollees accessed community care through the Choice Act.^9^ This study was approved by the Palo Alto VA research and development committee and the Stanford University institutional review board. A waiver of informed consent was granted because the study used secondary data. This study followed the Strengthening the Reporting of Observational Studies in Epidemiology (STROBE) reporting guideline.

### Data Sources

Patient demographic characteristics, health care utilization, and mortality data were obtained from the VA Corporate Data Warehouse (CDW), a repository of VA electronic medical records and enrollment and vital status data. We obtained community health care utilization from the CDW Fee Basis data and the Program Integrity Tool for VA Community Care data.

### Variable Definitions

The main independent variable in our analysis was a VA Choice Act eligibility indicator. Eligible individuals could be referred to a health care practitioner outside the VA in a network managed by contracted private insurance companies.^10^ Eligibility for the program was determined by wait time for a primary care appointment (≥30 days from the clinically determined date), unusual travel burden, or if their total travel distance to a VA facility was 40 or more miles. We focused only on the last criterion for the purpose of this study to ensure the most accurate identification of program eligibility. Distance from the VA facility was determined by the estimated driving distance using geocoded home address data by year.

We examined the rate of health care utilization by distance from the 40-mile travel distance threshold. We computed a rate by aggregating health care utilization for all enrollees who lived in 1-mile increments. To account for individuals moving residence, we looked within 1 calendar year and compiled utilization rates in location-year terms. Specifically, we took the numerator to be the number of visits for people living in that distance cell and the denominator to be the number of people who lived in that distance cell throughout the study period. In the case of inpatient visits and mortality, we multiplied the rate by 10 000 for readability.

Healthcare utilization was separately examined by outpatient and inpatient visits. Inpatient visits were characterized by any encounter in which the patient was admitted to a facility overnight (thus including observation stays). Postacute care, long-term care, and nursing home stays were excluded. All other encounters were classified as outpatient visits. To evaluate the association of the expanded network with patient decision-making, we excluded or separately analyzed some categories of visits that happen repeatedly. Specifically, we excluded chemotherapy and dialysis encounters, and we separately analyzed laboratory testing, home health aide and attendance, adult day care, telehealth encounters (telephone and video), occupational therapy, and individual and group psychotherapy. *Current Procedural Terminology *and Healthcare Common Procedure Coding System codes included in each category are listed in the eAppendix in the Supplement. Finally, we measured the number of medications individuals had on hand, ie, the number of active prescriptions that an individual had filled, as of June 1 of each year by using prescription fill dates and days’ supply of the fill.

### Statistical Analysis

We used an RD design, leveraging the sharp discontinuity in program eligibility that varies across the geographically determined distance threshold. In particular, we examined individuals who lived between 20 and 40 miles from a VA facility (approximately 2.7 million individuals) and compared the responses of veterans who lived than 40 miles from a VA facility with those who lived just less 40 miles from a VA facility. Full model specifications and regression tables are included in the eAppendix in the Supplement.

Next, it is possible that individuals took advantage of the VA’s expanded network and substituted 1 payer for another (eg, changed from Medicare or private insurance to the VA). To examine this, we looked only at VA enrollees who were dually enrolled in Medicare. This dual-enrollment group has a larger preexisting network of practitioners to choose from and, as a result, should have had less pent-up demand prior to the enactment of the Choice Act. If these individuals simply switched the payer for care they would have received anyway, then we would expect to see a drop in Medicare utilization corresponding to an increase in VA utilization at the 40-mile eligibility threshold. To determine this, we examined full Medicare claims data along with VA and VA community care data for individuals with dual enrollment from the period of 2015 to 2016 (the only years for which Medicare Advantage [MA] encounters were available). Health care practitioners who see patients enrolled in the VA in the community are required to accept Medicare rates, and as a result, we expected little to no change in the network size associated with the Choice Act for dual enrollees.

Under certain assumptions, the RD design minimizes the risk of confounders when examining the association of the Choice Act with utilization and mortality. First, eligibility should not be manipulable by the individual. This would be violated if a person moved or changed their address to make themselves eligible. We tested for this by including the number of individuals as the outcome variable in the RD regression.

The next assumption is that the cutoff was not systematically chosen such that individual-level factors that affected health outcomes would be associated with where around the distance threshold individuals resided. This assumption would be violated if, eg, the 40-mile threshold was selected to include a particular population, such as people with higher VA disability ratings. We determined this by estimating an RD model where the covariates—including age, race, disability rating, and Charlson Comorbidity Index—were the outcome of interest and to show that the RD estimates did not statistically differ from zero.

Finally, we conducted what is known as a placebo test by running the RD analysis using data from 2013, before the program started. As expected, this showed no association of utilization at the 40-mile threshold (eFigure 1 and eTable 1 in the Supplement). All analyses used 5% levels of significance, and hypothesis tests were 2-sided. Analyses were conducted in R version 4.0.5 (R Project for Statistical Computing). Data analysis was conducted from September 2020 to February 2021.

## Results

The study included more than 2.7 million unique individuals whose characteristics largely reflected the demographic characteristics of the VA system (mean [SD] age, 62 [17] years; 2 589 252 [90%] men; 282 168 [10%] Black; 2 203 352 [77%] White). Summary statistics of the sample are presented in Table 1. Enrollees who lived between 40 and 60 miles from the nearest VA facility were slightly older than those who lived between 20 and 39 miles away (mean [SD] age, 64 [16] years vs 62 [17] years). There was also a slightly higher mean (SD) proportion of White individuals (79.7% [41.1] vs 78.3% [42.0]), and a slightly lower mean (SD) proportion of individuals in priority groups 1 to 4 (47.2% [49.8] vs 49.1% [49.9]). Despite this, individuals living between 40 and 60 miles from the nearest VA facility had somewhat higher 2014 mean (SD) Charlson comorbidity scores (1.23 [0.25] vs 1.16 [0.24]). The plurality of enrollees were in priority groups 1 to 4, which implies at least 1 service-connected disability. These characteristics did not change abruptly with eligibility for the Choice Act, which is shown in the last column of Table 1, where no estimates were statistically significant. eTable 2 in the Supplement shows that the sample population was similar to the general VA population.

**Table 1.** Sample Characteristics and McCrary Test

Outcome	Proportion of patients by distance to nearest VA facility, mean (SD)		RD estimate	*P* value
	Mile 20-39	Mile 40-60		
Enrollees in 2014, No.	1 606 881	424 907	–6.878	.99
Patients with Medicare enrollment, 2014, No. (%)	946 897 (58.9)	276 024 (65.0)	–0.002	.54
Person-years, 2015-2018, No.	6 378 805	1 650 330	–4369	.40
Age, mean (SD), y	62.208 (16.683)	64.288 (15.961)	–0.04	.69
Race				
Black^a^	0.097 (0.292)	0.085 (0.278)	–0.001	.69
White^a^	0.783 (0.420)	0.797 (0.411)	0.005	.06
Sex				
Male	0.925 (0.273)	0.941 (0.246)	0.002	.11
Female	0.075 (0.263)	0.059 (0.236)		
Priority group				
1-4	0.491 (0.499)	0.472 (0.498)	–0.005	.12
5-7	0.301 (0.459)	0.307 (0.461)	0.001	.66
8	0.208 (0.409)	0.221 (0.418)	0.004	.16
Charlson Comorbidity Index score, mean (SD)	1.163 (0.237)	1.232 (0.251)	0.023	.09

Abbreviations: RD, regression discontinuity; VA, Veterans Affairs.

^a^ Black and White were the only 2 racial categories with sufficient sample size to estimate the McCrary test.

**Figure 1.**
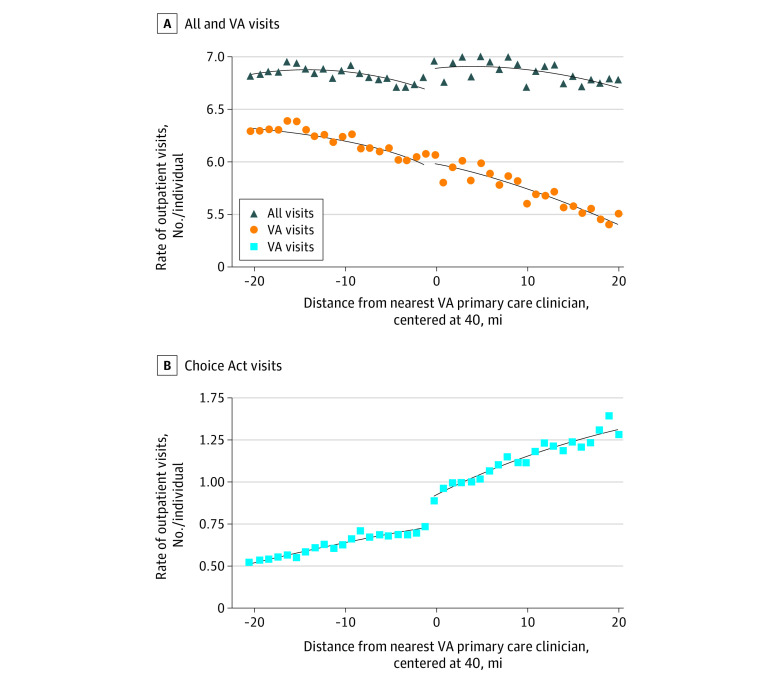
Change in Utilization by Distance to Nearest Veterans Affairs (VA) Primary Care Clinician Rate of outpatient utilization relative to the distance from the nearest VA primary care clinician. The rate is expressed as number of visits per person living in that distance cell from 2015 to 2018.

Figure 1 shows the rate of outpatient visits by distance from a VA facility from 2015 to 2018. Corresponding regression estimates are shown in Table 2. Outpatient visits increased significantly at the 40-mile threshold. Specifically, there was a 3.2% (95% CI, 1.0%-5.8%) increase in all visits, associated with a 24.3% (95% CI, 18.9%-29.6%) increase in Choice Act visits. Increases were the most concentrated among individuals with more service-connected disabilities and among younger individuals without service-connected disabilities, but not among those with high Charlson Comorbidity Index scores. Among patients in priority groups 1 to 4, outpatient visits increased by 3.1% (95% CI, 0.5%-5.7%); among individuals younger than 65 years in priority group 8, outpatient visits increased 7.0% (95% CI, 1.7%-12.3%). In contrast, individuals enrolled in Medicare did not increase outpatient visits in association with Choice Act eligibility (eFigure 2, eFigure 3, and eTable 3 in the Supplement).

**Table 2.** Change in Utilization at 40 Miles From Nearest VA Primary Care Clinician

Outcome	Enrollee group					
	All	High CCI	Priority group 1-4	<65 y, Priority group 8	PTSD	Medicare enrolled
Patient-years, No.	8 029 135	146 696	4 307 710	1 518 362	899 741	4 897 373
Outpatient						
Encounters per enrollee at mile 39, No.	6.7	15.5	8.5	0.8	12.9	7.7
Change in encounters per enrollee at mile 40, No. (95% CI)	0.23 (0.07 to 0.39)	0.30 (–0.06 to 0.65)	0.26 (0.04 to 0.48)	0.06 (0.01 to 0.10)	0.38 (0.14 to 0.63)	0.17 (–0.03 to 0.38)
Change in encounters per person at mile 40, No. (95% CI)	3.4 (1 to 5.8)	1.9 (–0.4 to 4.2)	3.1 (0.5 to 5.7)	7.0 (1.7 to 12.3)	3.0 (1.1 to 4.9)	2.3 (–0.4 to 4.9)
Outpatient, Choice Act						
Encounters per enrollee at mile 39, No.	0.7	1.8	0.9	0.1	1.3	0.9
Change in encounters per enrollee at mile 40, No. (95% CI)	0.18 (0.14 to 0.22)	0.31 (0.16 to 0.46)	0.23 (0.17 to 0.29)	0.04 (0.02 to 0.06)	0.32 (0.20 to 0.43)	0.17 (0.11 to 0.23)
Change in encounters per enrollee at mile 40, % (95% CI)	24.3 (18.9 to 29.6)	16.9 (8.8 to 25.0)	24.6 (18.0 to 31.1)	49.9 (28.4 to 71.4)	25.1 (15.8 to 34.3)	20.3 (13.3 to 27.3)
Inpatient						
Encounters per 10 000 enrollees at mile 39, No.	781.9	2579.3	936.8	86.7	1381.2	923.8
Change in encounters per 10 000 enrollees at mile 40, No. (95% CI)	9.5 (–19.4 to 38.3)	–30.2 (–132.9 to 72.5)	2.4 (–44.1 to 48.8)	1.9 (–13 to 16.9)	35.4 (–44.8 to 115.6)	1.6 (–36.3 to 39.4)
Change in encounters per 10 000 enrollees at mile 40, % (95% CI)	1.2 (–2.5 to 4.9)	–1.2 (–5.2 to 2.8)	0.3 (–4.7 to 5.2)	2.2 (–15.0 to 19.5)	2.6 (–3.2 to 8.4)	0.2 (–3.9 to 4.3)
Mortality						
Mortality rate per 10 000 enrollees at mile 39	346.0	575.5	315.7	56.2	223.5	498.5
Change in mortality rate per 10 000 enrollees at mile 40, No. (95% CI)	3.4 (–10.7 to 17.5)	7.4 (–21.6 to 36.4)	6.8 (–15.1 to 28.6)	–2.5 (–12.5 to 7.4)	8.8 (–15.2 to 32.7)	–0.7 (–17.4 to 16.1)
Change in mortality rate per 10 000 enrollees at mile 40, % (95% CI)	1.0 (–3.1 to 5.1)	1.3 (–3.8 to 6.3)	2.1 (–4.8 to 9.1)	–4.5 (–22.2 to 13.2)	3.9 (–6.8 to 14.6)	–0.1 (–3.5 to 3.2)

Abbreviations: CCI, Charlson Comorbidity Index; PTSD, posttraumatic stress disorder.

Total inpatient visits did not statistically significantly increase in association with the Choice Act (1.2%; 95% CI, –2.5% to 4.9%) (eFigure 4 and eTable 4 in the Supplement). This may be due to the Choice Act targeting nonemergent care or the naturally lower volume of inpatient visits. Additionally, we did not detect any evidence of changes to mortality with program eligibility. This was true overall and across all demographic groups and individuals with specific comorbidities.

We also examined the number of medications individuals took and the number of laboratory tests received (Table 3). As with outpatient visits, laboratory testing increased by 2.9% (95% CI, 1.0%-4.9%), while medications only increased by 1.2% (95% CI, 0.1%-2.3%). In contrast, individual and group psychotherapy increased substantially, with a 7.6% (95% CI, 2.5%-12.6%) increase overall and an 8.2% (95% CI, 1.7%-14.8%) increase for those with higher Charlson Comorbidity Index scores.

**Table 3.** Change in Utilization at 40 Miles from Nearest VA Primary Care Clinician for Specific Categories of Outpatient Procedures

Outcome	Enrollee group					
	All	High CCI	Priority group 1-4	Under 65, priority group 8	PTSD	Medicare enrolled
Patient-years, No.	8 029 135	146 696	4 307 710	1 518 362	899 741	4 897 373
Psychotherapy^a^						
Encounters per enrollee at mile 39, No.	0.4	0.5	0.6	0.1	1.8	0.3
Change in encounters per enrollee at mile 40, No. (95% CI)	0.03 (0.01 to 0.05)	0.04 (0.01 to 0.07)	0.05 (0.01 to 0.08)	0.00 (–0.01 to 0.01)	0.11 (0.04 to 0.19)	0.03 (0.01 to 0.05)
Change in encounters per person at mile 40, % (95% CI)	7.6 (2.5 to 12.6)	8.2 (1.7 to 14.8)	7.6 (2.0 to 13.2)	–1.7 (–21.0 to 17.7)	6.4 (2.1 to 10.6)	7.7 (1.5 to 13.9)
Telehealth^b^						
Encounters per enrollee at mile 39	1.6	3.6	1.8	0.2	2.8	1.8
Change in encounters per enrollee at mile 40, No. (95% CI)	0.01 (–0.01 to 0.04)	0.00 (–0.05 to 0.06)	0.01 (–0.04 to 0.05)	0.01 (–0.01 to 0.03)	0.00 (–0.05 to 0.05)	0.00 (–0.03 to 0.03)
Change in encounters per enrollee at mile 40, % (95% CI)	0.8 (–0.9 to 2.5)	0.1 (–1.3 to 1.6)	0.3 (–2.3 to 2.9)	5.7 (–2.7 to 14.0)	–0.1 (–1.9 to 1.8)	0.0 (–1.6 to 1.7)
Home health^c^						
Encounters per 10 000 enrollees at mile 39	13.2	30.9	16.7	1.4	24.2	15.6
Change in encounters per 10 000 enrollees at mile 40, No. (95% CI)	0.39 (0.15 to 0.64)	0.45 (–0.01 to 0.91)	0.36 (0.06 to 0.66)	0.10 (0.03 to 0.17)	0.41 (–0.08 to 0.91)	0.29 (–0.02 to 0.60)
Change in encounters per 10 000 enrollees at mile 40, % (95% CI)	3.0 (1.1 to 4.8)	1.5 (0 to 3.0)	2.1 (0.4 to 3.9)	6.9 (2.1 to 11.8)	1.7 (–0.3 to 3.7)	1.9 (–0.1 to 3.8)
Laboratory tests						
Encounters per enrollee at mile 39	1.6	3.4	1.8	0.3	2.4	1.9
Change in encounters per enrollee at mile 40, No. (95% CI)	0.05 (0.02 to 0.08)	0.05 (–0.02 to 0.11)	0.05 (0.01 to 0.09)	0.02 (0 to 0.03)	0.03 (–0.02 to 0.09)	0.04 (0 to 0.08)
Change in encounters per enrollee at mile 40, % (95% CI)	2.9 (1.0 to 4.9)	1.4 (–0.5 to 3.2)	2.7 (0.5 to 4.8)	6.0 (–1.4 to 13.4)	1.4 (–1.0 to 3.9)	2.2 (0.3 to 4.2)
No. of perscriptions^d^						
Prescriptions per enrollee at mile 39, No.	3.2	7.1	3.9	1.2	6.1	4.0
Change in prescriptions per enrollee at mile 40, No. (95% CI)	0.04 (0 to 0.07)	0.01 (–0.08 to 0.07)	0.06 (0 to 0.12)	0.06 (–0.01 to 0.12)	0.01 (–0.08 to 0.1)	0.0 (–0.06 to 0.06)
Change in prescriptions per enrollee at mile 40, % (95% CI)	1.2 (0.1 to 2.3)	0.0 (–1.1 to 1.0)	1.5 (0 to 3.0)	4.7 (–0.9 to 10.3)	0.2 (–1.2 to 1.7)	0.0 (–1.4 to 1.5)

Abbreviations: CCI, Charlson Comorbidity Index; PTSD, posttraumatic stress disorder.

^a^ Psychotherapy includes individual and group therapy.

^b^ Telehealth includes telephone and video evaluation and management visits.

^c^ Home health includes home health, adult day care, and aid and attendance visits.

^d^ The prescription counts are the mean number of active medications on hand for each person as of June 1 of each year from 2015 to 2018.

**Figure 2.**
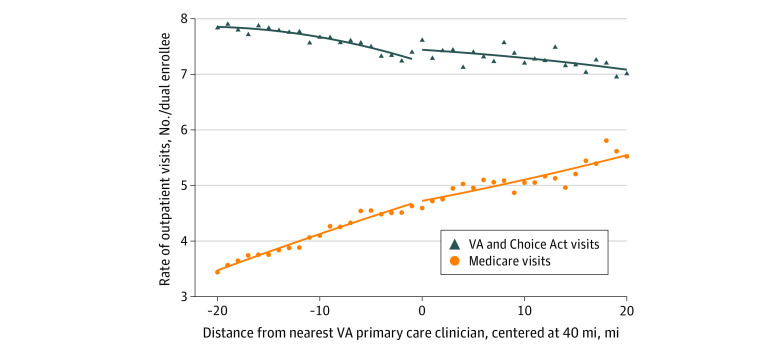
Location Profile of Outpatient Visits for Patients Dually Enrolled in Veterans Affairs (VA) and Medicare Rate of outpatient visits by distance from nearest VA primary care clinician per VA enrollee location-year with data from 2015 to 2016. Medicare includes traditional Medicare and Medicare Advantage.

Figure 2 shows that there was no change to the rate of Medicare visits at the discontinuity among dually enrolled individuals in 2015 to 2016. It also shows that overall outpatient utilization for Medicare dual enrollees appears to have increased slightly, but the estimate was not statistically significant (3.1%; 95% CI, –0.2% to 6.5%) (eTable 5 in the Supplement).

## Discussion

This is one of few studies, to our knowledge, to examine how the expansion of health care networks for those with existing coverage is associated with changes in health care utilization and outcomes. Our analysis is distinct in that we examined changes to network size without the presence of common confounders, such as varying monthly premiums and shifts in patient population (ie, patient selection). The results can also be used to ascribe the value of expanded networks (ie, through revealed patient preferences). We found a moderately large response to an expansion in network access, implying that the value of these additional options is likely high to individuals and, thus, their loss may be costly. We also found that Medicare visits for dual enrollees did not change significantly in association with Choice Act eligibility, suggesting that the overall outpatient utilization increase with Choice Act eligibility was not driven by patient substitution across payers. Furthermore, this is the first study to show how VA community care is associated with health care utilization and mortality for VA enrollees, which is significant for both VA’s ongoing efforts to ensure timely, high-quality health care for veterans and for VA’s budgetary planning and resource allocation.

### Policy Implications for Insurance Coverage Networks

Employers and insurance companies are embracing narrow network plans as a way to save costs. In the context of ACA marketplaces, it is one of the few mechanisms available to insurers to reduce premiums. However, narrow networks can pose problems for access to care, such as longer wait times and challenges in finding specialists.^11^ Narrow networks have been offered as a source of potential savings for runaway health care costs in the United States in reasonably competitive markets.^1,2^ It has been shown that consumers are willing to pay for a broader network, but it is generally unknown whether that implies preferences for quality, choice, or individual clinicians.^4,7^

However, examining and determining the optimal network size is not solely an issue with private commercial insurance plans. MA plans often offer a narrower network than traditional Medicare and typically cost less than a traditional Medicare plan with Medigap and Part D added. The drawback to consumers is that they are restricted to seeing in-network practitioners. Because MA plans offer the same benefits as traditional Medicare, previous research has shown that cost savings are derived from managing enrollee utilization along with the quality of included clinicians.^12,13,14^ Medicaid has also been characterized as a narrow-network plan for certain services in some locations.^15,16,17^

This study adds to previous evidence that network size modulates health care consumption.^3,14,18^ We found associations that were strikingly similar in magnitude to previous work addressing decreases in network size, with previous work in private insurers showing a 2% to 3% decrease in utilization.^3,18^ However, our estimates are far smaller than a previous study that found a 60% increase in inpatient stays from a network expansion caused by MA patients being forced into traditional Medicare.^14^ There are a few reasons this may be the case. First, expanding VA’s network was substantially slower than those previously studied. VA enrollees had to be aware of the program, check their eligibility, and find local practitioners willing to accept VA patients.^19^ Second, a relatively smaller portion of our cohort may have been directly affected. Unlike changes to private insurance networks that affect all enrollees in that plan, only 20% of VA enrollees report no other forms of health insurance coverage.^20^ Finally, our estimates necessarily focused on individuals who live close to the distance threshold. This means individuals in this study were less likely to live in urban areas, and it is likely that there exists a strong gradient in treatment effects of network size.

Most of the research on network size has focused on utilization and/or spending, but increased utilization may be beneficial (ie, if there is pent-up demand from inadequate access) or wasteful (ie, if an expanded network leads to low-value care). Clearly, the latter is much more problematic when care is provided in high-cost settings. However, drawing such conclusions would require us to determine the value of the care, and determining medical necessity was beyond the scope of this article. Also, while we did not detect changes to mortality, future work should focus on determining whether expanded networks are associated with end points, such as readmissions, that positively affect patient well-being.

### Policy Implications for VA Health Care System

By expanding access, the Choice Act was associated with an overall increased utilization of 3.2%. The implications of this are important for the VA from a longer-term budgetary sense. Efficient allocation of staff and facilities becomes more difficult when fragmented between purchased and provided care, and the VA has required emergency congressional funding because of underestimates in predicting the use of community care.^21^ Without larger budgets, the VA may struggle to sustain its current infrastructure of hospitals and outpatient clinics while also providing more community care. This is especially pressing given that the Maintaining Internal Systems and Strengthening Integrated Outside Networks (MISSION) Act of 2019 further expanded access to community care.^22^

### Limitations

There are limitations to this study. First, we cannot elicit the exact reasons patients decided to use community care nor can we determine the medical necessity for visits. Second, it is possible that people who lived less than 40 miles from a VA facility accessed community care if they were subject to long wait times or hardships; this would bias the results toward the null. Third, while a benefit of the RD design is to eliminate confounders, it limits the analysis to examining individuals living close to the 40-mile threshold. Thus, it is possible that our results do not generalize to different distance thresholds such that there may be different outcomes for patients that live closer to (or farther from) a VA facility. Fourth, even though most VA enrollees also use non-VA practitioners, it is possible that the results from VA enrollees may not be generalizable to the general population.

## Conclusions

We examined the VA Choice Act and contributed to the discussion of network access by examining how expansions in networks are associated with health care utilization and outcomes. Our findings suggest that expanded access can lead to increases in utilization across health care categories and patient demographic characteristics. However, further work is needed to determine the sustainability and efficiency of the increases in utilization.

## Notes

Supplement. **eAppendix. **Supplemental Material **eFigure 1. **Location Profile of Outpatient Visits, CY2013 **eTable 1. **Change in Outpatient at 40 Miles From Nearest VA Primary Care Clinician in CY2013, Before the Choice Act Was Implemented **eTable 2. **Summary Statistics by Where Individuals Lived in 2014 Prior to the Choice Act **eFigure 2. **Bandwidth Robustness of the Change in Outpatient Visits at the 40-Mile Threshold **eFigure 3. **Location Profile of Mortality **eTable 3. **Change in Outpatient Utilization at 40 Miles From Nearest VA Primary Care Clinician **eFigure 4.** Location Profile of Inpatient Visits **eTable 4. **Change in Inpatient Utilization at 40 Miles from Nearest VA Primary Care Clinician **eTable 5. **Outpatient Visits for Patients With Dual Enrollment in VA and Medicare
