# Error in Figure

**DOI:** 10.1001/jamanetworkopen.2021.40139

**Published:** 2021-11-19

In the Original Investigation titled “Association of Expanded Health Care Networks With Utilization Among Veterans Affairs Enrollees,”^1^ published October 26, 2021, there was an error in Figure 1. An incorrect version of the figure was published. This article has been corrected.^1^
