# Supplementary material for: Association of Expanded Health Care Networks With Utilization Among Veterans Affairs Enrollees
Source: JAMA Netw Open. 2021 Oct 26;4(10):e2131141. doi: 10.1001/jamanetworkopen.2021.31141 (PMC8548943; doi:10.1001/jamanetworkopen.2021.31141)
Supplement: Supplement. — eAppendix. Supplemental Material eFigure 1. Location Profile of Outpatient Visits, CY2013 eTable 1. Change in Outpatient at 40 Miles From Nearest VA Primary Care Clinician in CY2013, Before the Choice Act Was Implemented eTable 2. Summary Statistics by Where Individuals Lived in 2014 Prior to the Choice Act eFigure 2. Bandwidth Robustness of the Change in Outpatient Visits at the 40-Mile Threshold eFigure 3. Location Profile of Mortality eTable 3. Change in Outpatient Utilization at 40 Miles From Nearest VA Primary Care Clinician eFigure 4. Location Profile of Inpatient Visits eTable 4. Change in Inpatient Utilization at 40 Miles from Nearest VA Primary Care Clinician eTable 5. Outpatient Visits for Patients With Dual Enrollment in VA and Medicare [file jamanetwopen-e2131141-s001.pdf]

Supplemental Online Content

Rose L, Aouad M, Graham L, Schoemaker L, Wagner T. Association of expanded health care networks with utilization among Veterans Affairs enrollees. *JAMA Netw Open.* 2021;4(10):e2131141. doi:10.1001/jamanetworkopen.2021.31141

eAppendix. Supplemental Material

eFigure 1. Location Profile of Outpatient Visits, CY2013

eTable 1. Change in Outpatient at 40 Miles From Nearest VA Primary Care Clinician in CY2013, Before the Choice Act Was Implemented

eTable 2. Summary Statistics by Where Individuals Lived in 2014 Prior to the Choice Act

eFigure 2. Bandwidth Robustness of the Change in Outpatient Visits at the 40-Mile Threshold

eFigure 3. Location Profile of Mortality

eTable 3. Change in Outpatient Utilization at 40 Miles From Nearest VA Primary Care Clinician

eFigure 4. Location Profile of Inpatient Visits

eTable 4. Change in Inpatient Utilization at 40 Miles from Nearest VA Primary Care Clinician

eTable 5. Outpatient Visits for Patients With Dual Enrollment in VA and Medicare

This supplemental material has been provided by the authors to give readers additional information about their work.

© 2021 Rose L et al. *JAMA Network Open.*

eAppendix. Supplemental Material

Technical Appendix

This appendix provides more information about the regression discontinuity design. Regressions are estimated using collapsed data at the distance in miles cell level. The estimating equation is:

$$Y_d = \alpha_0 + \beta Over40_d + \alpha_1 D_d + \alpha_2 D_d * Over40_d + \alpha_3 D_d^2 + \alpha_4 D_d^2 * Over40_d^2 + \varepsilon_d$$

In this model, Y_d is the rate of visits or deaths per location years for the one mile location cell d, $Over40_d$ is an indicator for being over the 40-mile threshold, and D_d is distance to VA re-centered at 40 miles. Regressions were weighted by the number of enrollees in each location cell, and the number of eligible enrollees in the case of heterogeneity analyses (e.g. the number of Medicare enrolled individuals). We use a quadratic polynomial, but results are robust to linear or local linear approaches. We also perform the analysis separately for different subsamples, focusing on heterogeneous responses across different demographic groups, defined prior to the start of the program. Additionally, optimal bandwidth procedures suggest bandwidths between 18-25 miles depending on the outcome and subsample; we use a 20 mile bandwidth for simplification, but show bandwidth robustness checks in Supplementary Exhibit A1.

Encounters were considered inpatient visits if they occurred at an acute care hospital and were coded as inpatient in VA or community claims records. Visits were counted as lab visits if they had an associated CPT code for laboratory services (80000-89999) or venipuncture for specimen collection (36592). Psychotherapy visits were coded with CPTs 90832-90853 and 90863. Home health, adult day care, and aid and attendance were coded with the following CPT and HCPCS codes: G0156, G0162, G0299, G0300, G0493-G0496, S9123, S9124, T1000, T1030, S5100-S5105, S5108-S5111, S5115, S5116, S5120-S5151, S5170, S9098, S9222, T1004, T1019-T1021. Physical therapy and occupational therapy were coded with the following CPT and HCPCS codes: G1051-G0161, G0168, G2168, G2169, 97161, 97162, 97110, 97012, 97530, 98940-98943.

© 2021 Rose L et al. *JAMA Network Open.*

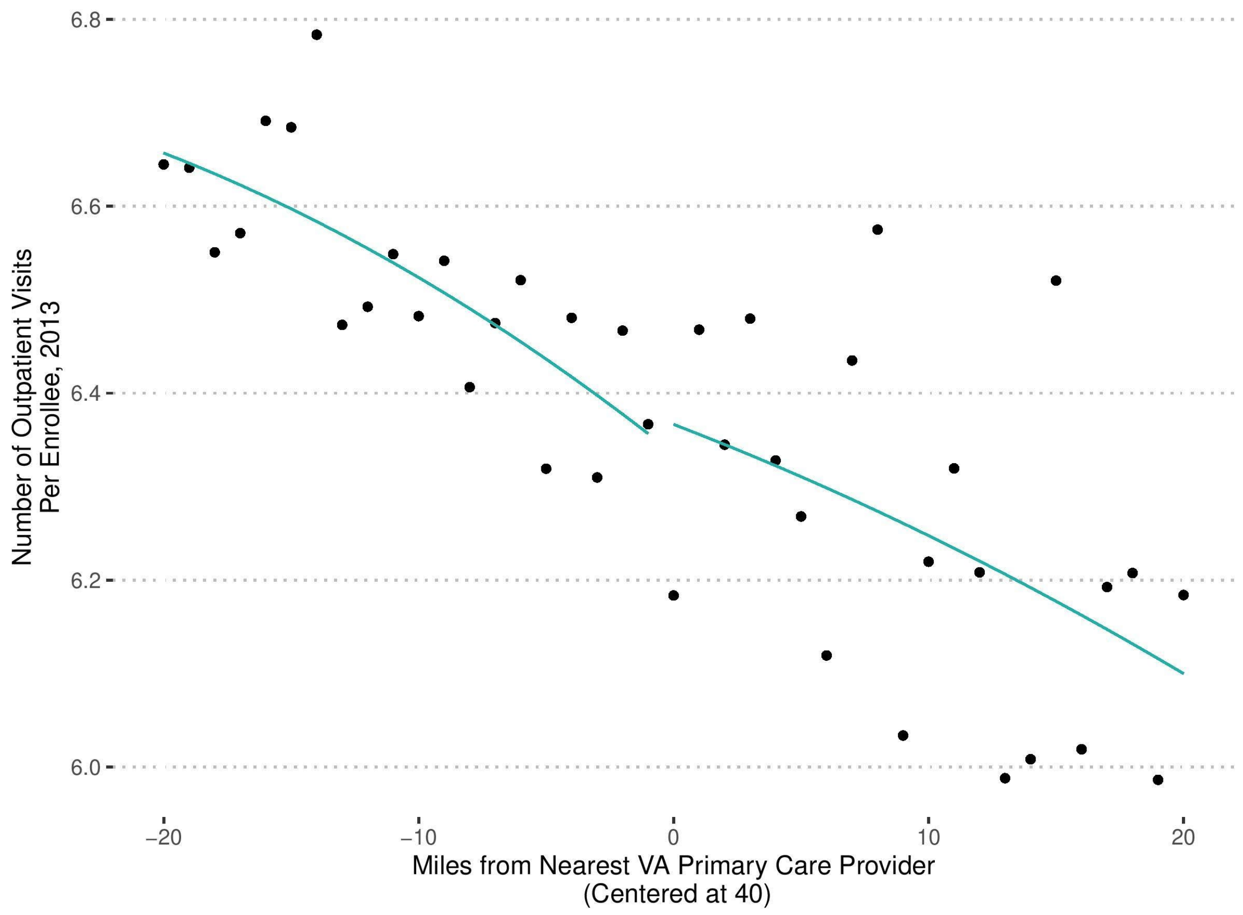

Source/Notes: Author analysis of VA data. NOTES Number of outpatient visits by distance from nearest VA primary care provider per VA enrollee location-years with data from 2013, before the Choice Act was implemented.

© 2021 Rose L et al. *JAMA Network Open.*

eTable 1. Change in Outpatient at 40 Miles From Nearest VA Primary Care Clinician in CY2013, Before the Choice Act Was Implemented	
Outcome	All Enrollees
Outpatient - Overall	0.002
	(0.099)
	[7.22]

Source/Notes: Author analysis of VA data. NOTES Results from a regression of the change in the rate of mortality at 40 miles from the closest VA primary care provider in 2013. Regression results are shown as regression estimate, the standard error in parentheses, and the estimate of the level of utilization just before the threshold in brackets.

**eTable 2.** Summary Statistics by Where Individuals Lived in 2014 Prior to the Choice Act			
Distance to VA (2014)	<20 Miles	>60 Miles	20-60 Miles (Study Sample)
Enrollees	5489389	121039	2031788
Age	61.1	64.9	62.6
White	0.68	0.81	0.79
Black	0.18	0.03	0.09
Male	0.91	0.94	0.93
Priority Group 1-4	0.48	0.46	0.49
Priority Group 5-7	0.32	0.31	0.30
Priority Group 8	0.20	0.24	0.21
Charlson Comorbidity Index	1.15	1.17	1.18
Medicare Enrolled	2945406	79449	1222921

© 2021 Rose L et al. *JAMA Network Open.*

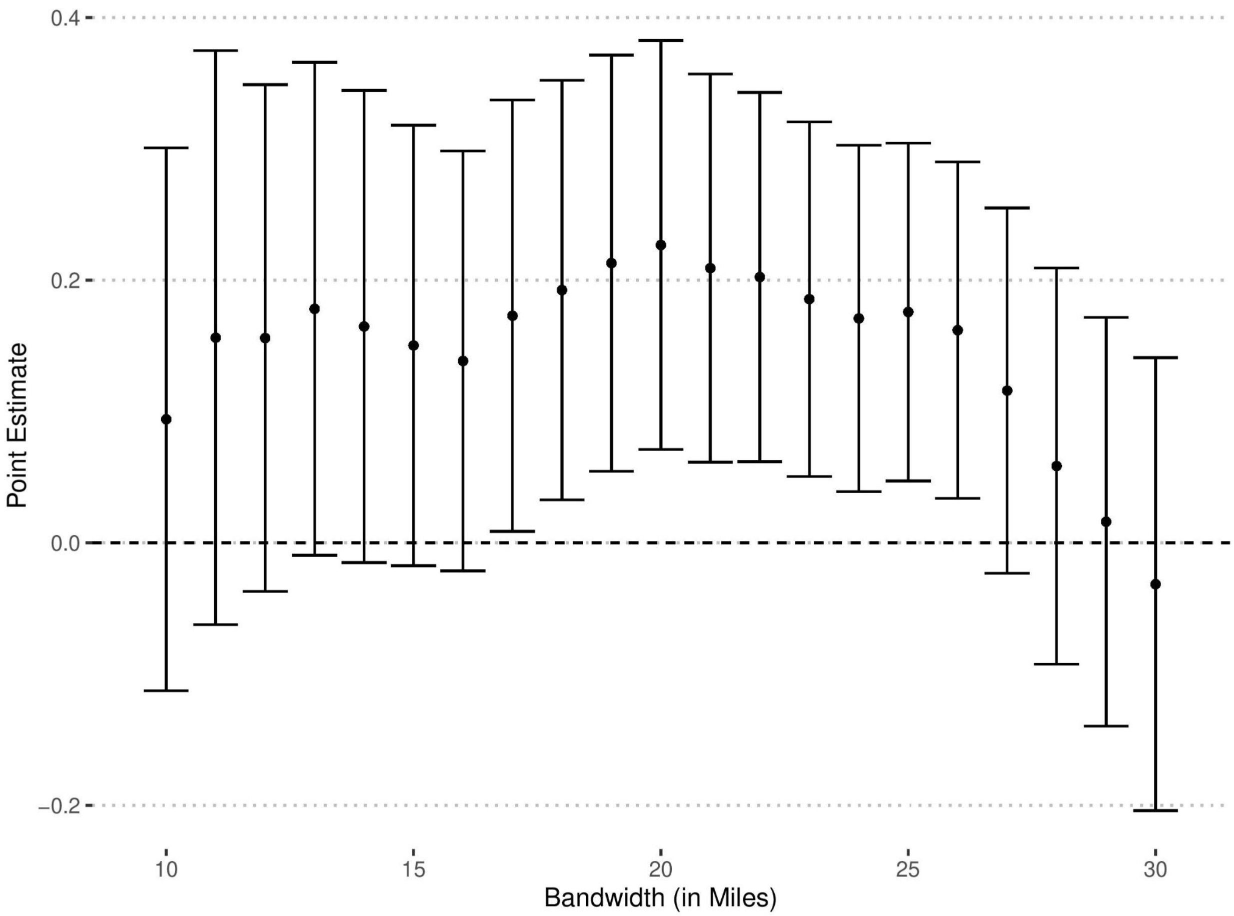

eFigure 2. Bandwidth Robustness of the Change in Outpatient Visits at the 40-Mile Threshold

Notes: Each point is a regression estimate of the change in the rate of outpatient utilization at 40 miles from the closest VA primary care provider. Points are shown with 95% error bars.

© 2021 Rose L et al. *JAMA Network Open.*

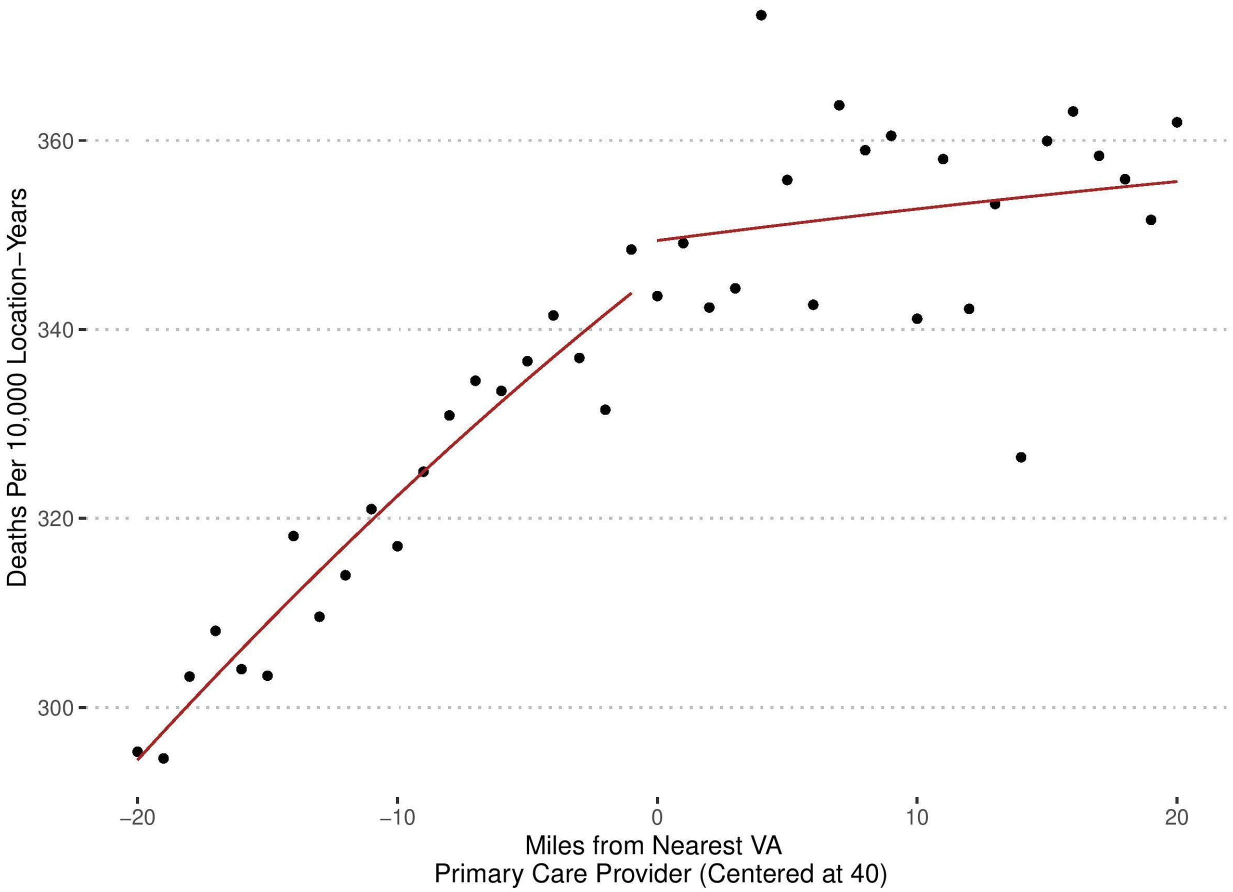

eFigure 3. Location Profile of Mortality

Source/Notes: Author analysis of VA data. NOTES Deaths by distance from nearest VA primary care provider per 10,000 VA enrollee location-years with data from 2015-2018.

© 2021 Rose L et al. *JAMA Network Open.*

eTable 3. Change in Outpatient Utilization at 40 Miles From Nearest VA Primary Care Clinician

Outcome	All Enrollees	High CCI	Priority Group 1-4	Under 65, Priority Group 8	PTSD	Medicare Enrolled
Outpatient - Overall	0.23	0.3	0.26	0.06	0.38	0.17
	(0.07-0.39)	(-0.06-0.65)	(0.04-0.48)	(0.01-0.1)	(0.14-0.63)	(-0.03-0.38)
	[6.7]	[15.5]	[8.5]	[0.8]	[12.9]	[7.7]
Outpatient - Choice	0.18	0.31	0.23	0.04	0.32	0.17
	(0.14-0.22)	(0.16-0.46)	(0.17-0.29)	(0.02-0.06)	(0.2-0.43)	(0.11-0.23)
	[0.7]	[1.8]	[0.9]	[0.1]	[1.3]	[0.9]
Outpatient - VA	0.05	-0.01	0.03	0.01	0.07	0
	(-0.13-0.22)	(-0.43-0.41)	(-0.19-0.26)	(-0.02-0.05)	(-0.21-0.34)	(-0.22-0.22)
	[5.9]	[13.6]	[7.6]	[0.7]	[11.6]	[6.8]

NOTES Results from a regression of the change in the rate of outpatient utilization enrollees at 40 miles from the closest VA primary care provider. Regression results are shown as regression estimate, 95% confidence interval, and the estimate of the level of utilization just before the threshold in brackets. *VA Outpatient* refers to care provided at VA facilities, and *Choice Outpatient* refers to care provided through the Choice Program.

© 2021 Rose L et al. *JAMA Network Open.*

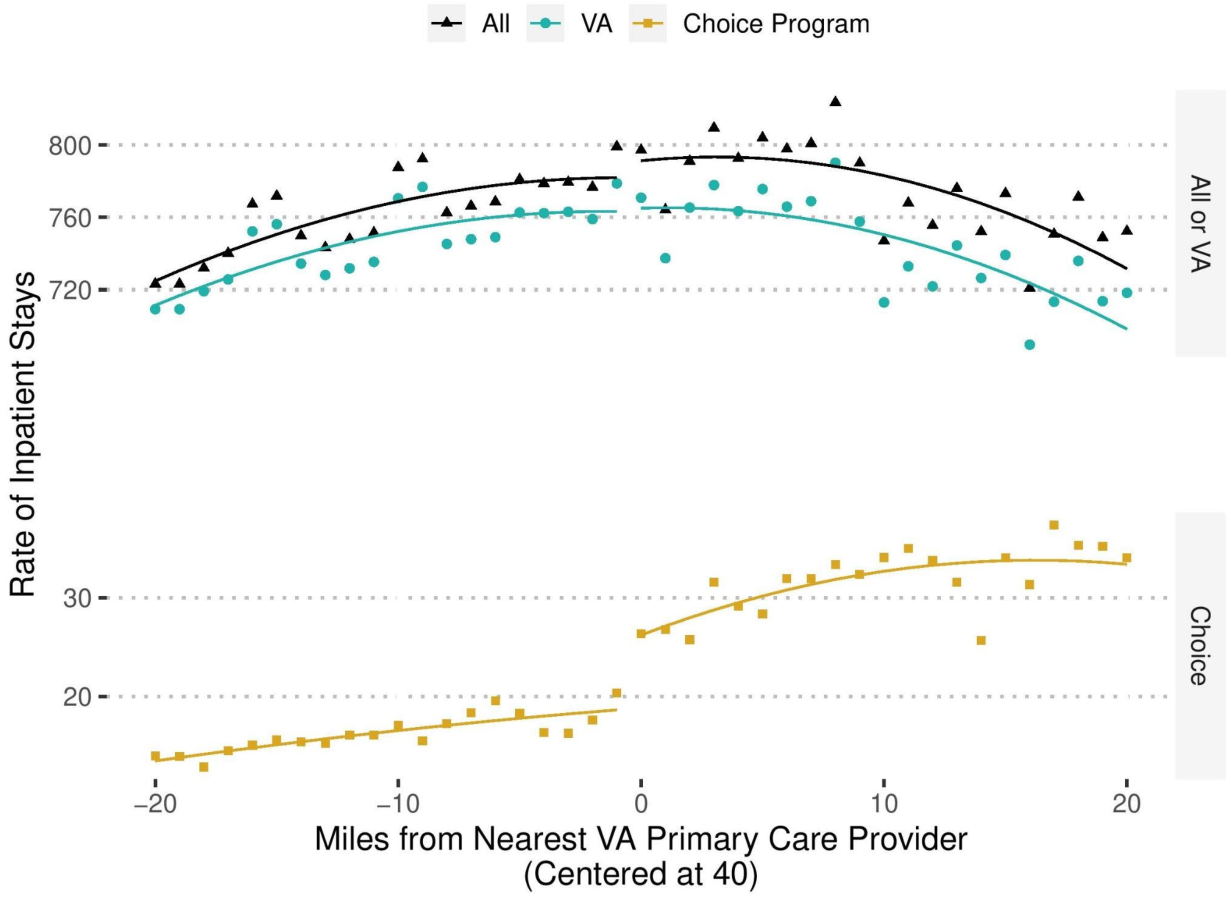

eFigure 4. Location Profile of Inpatient Visits

Source/Notes: Author analysis of VA data. NOTES Number of inpatient visits by distance from nearest VA primary care provider per VA enrollee location-years with data from 2015-2018. Black triangles denote all outpatient visits, blue circles denote VA outpatient visits, and yellow squares denote outpatient visits with the VA Choice program.

© 2021 Rose L et al. *JAMA Network Open.*

eTable 4. Change in Inpatient Utilization at 40 Miles from Nearest VA Primary Care Clinician						
Outcome	All Enrollees	High CCI	Priority Group 1-4	Under 65, Priority Group 8	PTSD	Medicare Enrolled
Inpatient - All	9.5	-30.2	2.4	1.9	35.4	1.6
	(-19.4-38.3)	(-132.9-72.5)	(-44.1-48.8)	(-13-16.9)	(-44.8-115.6)	(-36.3-39.4)
	[781.9]	[2579.3]	[936.8]	[86.7]	[1381.2]	[923.8]
Choice Inpatient	7.4	12.2	7	2	9.7	8
	(4.7-10.1)	(6.7-17.7)	(3.6-10.5)	(0.3-3.7)	(0.9-18.6)	(4.5-11.6)
	[18.8]	[36.3]	[23]	[1.7]	[29.9]	[21.9]
VA Inpatient	2.1	-42.4	-4.7	-0.1	25.7	-6.5
	(-25.7-29.8)	(-145.6-60.8)	(-48.8-39.5)	(-14.4-14.3)	(-56.3-107.6)	(-42.9-30)
	[763.1]	[2543]	[913.8]	[85]	[1351.3]	[901.9]

NOTES Results from a regression of the change in the rate of inpatient utilization per 10,000 enrollees at 40 miles from the closest VA primary care provider. Regression results are shown as regression estimate, 95% confidence interval, and the estimate of the level of utilization just before the threshold in brackets. *VA Inpatient* refers to care provided at VA facilities, and *Choice Inpatient* refers to care provided through the Choice Program.

© 2021 Rose L et al. *JAMA Network Open.*

eTable 5. Outpatient Visits for Patients With Dual Enrollment in VA and Medicare

Outcome	VA+Choice	Medicare
Change in encounter rate per enrollee at mile 40 (95% CI)	0.22 (-0.02-0.46)	-0.01 (-0.19-0.17)
Encounter rate per enrollee at mile 39	[7.21]	[4.73]
% Change in encounter rate per enrollee at mile 40 (95% CI)	3.1 (-0.2-6.5)	-1.9 (-3.9-3.6)

Notes: Regression results are shown as the change in the rate of encounters at 40 miles, 95% confidence interval, the rate of encounters just before the 40-mile threshold in brackets, and the percent change in the rate followed by the 95% confidence interval.

© 2021 Rose L et al. *JAMA Network Open.*
